# Coved and Saddleback ST-Segment Elevations: Brugada Phenocopy vs Brugada Syndrome

**DOI:** 10.7759/cureus.24338

**Published:** 2022-04-21

**Authors:** Jocelyn McCullough, Joseph McCullough, Marcella Gonzalez

**Affiliations:** 1 Medical Education, Zucker School of Medicine, Hempstead, USA; 2 Hospital Medicine, Zucker School of Medicine, Hempstead, USA

**Keywords:** non-structural cardiac conduction abnormality, cardiac sudden death, reversible ecg finding, brugada syndrome, brugada ecg pattern

## Abstract

We report a case of a middle-aged man who presented with near syncope, fever, and dysuria and was incidentally found to have coved ST-segment elevations in leads V1 and V2 confirming Brugada type 1 ECG (electrocardiogram) pattern. This ECG pattern morphed into saddleback ST-segment elevations in precordial leads consistent with type 2 Brugada the following day. Additionally, the patient reported a positive family history of sudden cardiac death. This initial presentation made it impossible to differentiate Brugada phenocopy (BrP) from Brugada syndrome (BrS). Continuous cardiac monitoring was initiated, electrophysiology consulted and fever managed with antipyretics. The patient was diagnosed with prostatitis and bacteremia from E. coli and managed with antibiotics. There were no electrolyte abnormalities nor was the patient on any medications other than tamsulosin for his chronic benign prostate hypertrophy. Once the fever resolved the patient's ECG returned to normal, thus confirming the diagnosis of BrS on day 3 post-admission. Differentiating between BrP and BrS requires ruling out possible underlying causes and determining if resolution in ECG patterns occurs.

## Introduction

Over 70% of patients with BrS do not have an inherited disorder and lack known genetic mutations, but up to 30% may have spontaneous mutations without a positive family history of sudden cardiac death. The most commonly recognized genetic mutation for BrS is inherited as an autosomal dominant mutation in the beta subunit of the sodium channel of SCN5A gene [1-4]. This mutation has been associated with defective cardiac conduction causing life-threatening ventricular arrhythmias. Other genetic mutations associated with BrS include: CACN1Ac gene encoding for the alpha subunit of cardiac calcium channels 2,3,4, SCN1B2,3, SCN2B2, SCN3B2,3, GPD1-L2,3, HEY22, PKP22, RANGRF2, SCN10A2, SLMAP2, and TRPM42. The ECG abnormalities seen in this condition are ST-segment elevations of >2 mm in precordial leads V1 and V2 in the absence of structural cardiac abnormalities. Brugada syndrome is more common in men above 40 and is 8-10 times more prevalent than women, mostly seen in the Southeast Asian population. On the other hand, Brugada phenocopy (BrP) is an acquired disorder that can be precipitated by various clinical conditions. BrP also involves the presence of ECG findings similar to BrS but these ECG findings normalize after treating the underlying cause and there is no family history of sudden cardiac death among these patients [5-9]. While it is difficult to initially differentiate the two, a correct diagnosis is important to prevent life-threatening arrhythmias and sudden cardiac death with BrS but not in patients with BrP.

## Case presentation

A 63-year-old Spanish-speaking male with a history of benign prostatic hyperplasia presented to the hospital with dysuria and fever for three days. During initial history taking, the patient stated that the prior night he felt weak while he was walking to the restroom and reported symptoms of lightheadedness and had a near syncopal event that resolved within five minutes, after sitting down. The patient was only on tamsulosin and did not report the use of any alcohol or recreational drugs. Upon arrival at the hospital, the patient was febrile with a temperature of 102.9 F, tachycardic with a heart rate of 107, and elevated blood pressure of 144/82 mmHg. On physical examination, the patient was alert and oriented to time, person, and place. He had normal S1 and S2 heart sounds without any murmurs, rubs, or gallops. His abdominal exam was non-distended without any supra-pubic or CVA (costovertebral angle tenderness). The prostate exam was significant for tenderness to palpation. 

**Figure 1:**
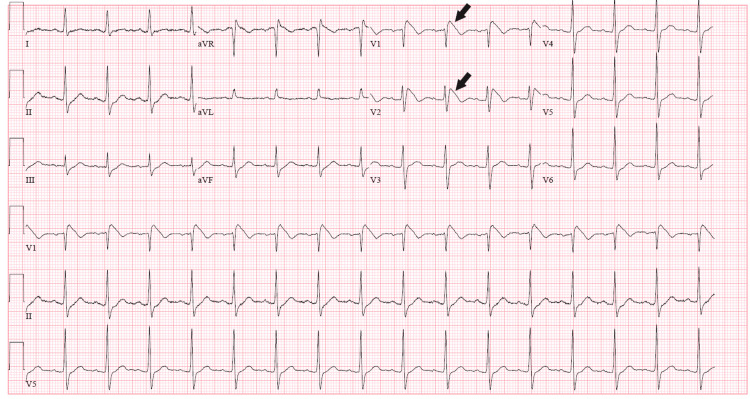
Day 1 ECG showed coved ST segment elevations in leads V1 and V2 (black arrows)

The patient’s basic metabolic panel did not reveal any electrolyte abnormalities, his CBC showed leukocytosis of 14.99 K/µL (3.8-10.5 K/µL, and lactate of 2.5 mmol/L (0.5-1 mmol/L) and a urine analysis demonstrated bacteriuria and pyuria. Computed tomography (CT) with IV contrast (iohexol) showed inflammation of the bladder wall and prostate enlargement. The patient was diagnosed with sepsis secondary to prostatitis and given 2.5 liters lactated ringers boluses and ceftriaxone 1 g IV daily. He also received acetaminophen for fever. The patient’s ECG incidentally showed coved ST segments in precordial leads V1 and V2, suggestive of Brugada type 1 pattern (Figure 1). Troponin from admission was <0.01ng/mL (<0.04ng/mL). A transthoracic echocardiogram showed a normal left ventricular ejection fraction of 65% without any other structural abnormalities. Electrophysiology and cardiology were consulted and recommended admission for continuous cardiac monitoring. Upon further questioning, the patient denied ongoing chest pain or palpitations but revealed a history of sudden cardiac death of a first-degree relative.

On day 2 of admission the urine culture and blood cultures were positive in both aerobic and anaerobic bottles to E. coli and based on antibiotic sensitivity Meropenem 1 g IV every eight hours was initiated instead of ceftriaxone.

**Figure 2:**
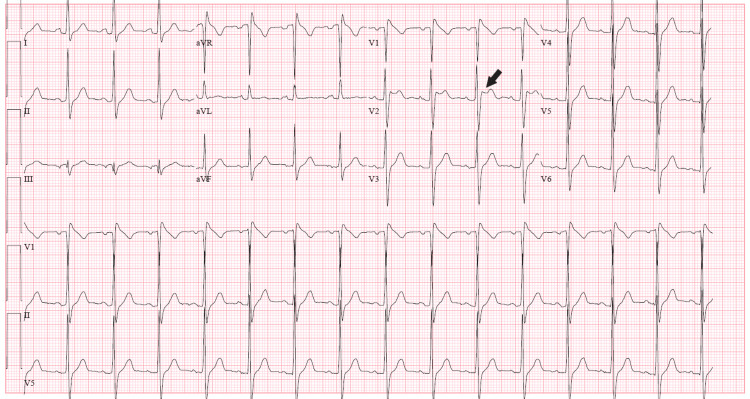
Day 2 ECG showed saddleback ST segment elevations in lead V2 (black arrow)

Repeat ECG, as recommended by the electrophysiology team, showed saddleback pattern of ST segments in leads V1/V2 demonstrating Brugada type 2 pattern (Figure 2). Repeat troponin was <0.0ng/mL. Electrophysiology recommended that any additional medications be checked prior to administration on www.brugadadrugs.org to prevent provoking life-threatening arrhythmia and sudden cardiac death.

**Figure 3:**
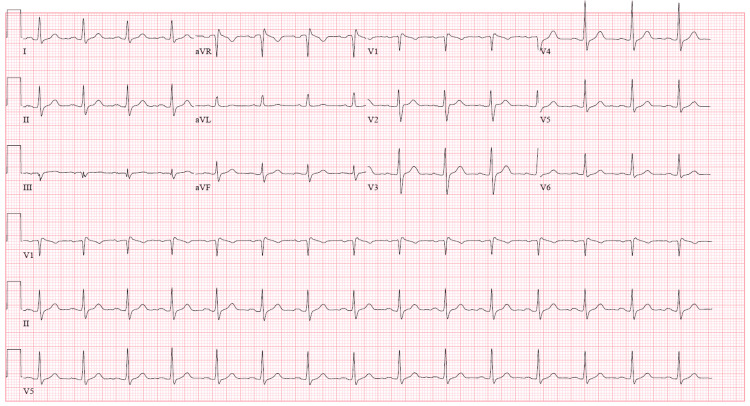
Day 3 ECG showed normalization of ST segment elevations in precordial leads

Repeat EKG on day 3 of admission showed complete resolution of all Brugada type patterns (Figure 3). The patient was discharged on day 3 and switched to levofloxacin for another four weeks. The patient followed up with a cardiologist two weeks post discharge and repeat ECG in the office revealed normalization of the ST elevations confirming BrS induced by fever and is currently awaiting provocative test with sodium channel blockers (flecainide, ajmaline, or procainamide). The patient was instructed to seek medical attention in the emergency room if fever greater than 100.4 F did not abate with antipyretics. He was also given a list of medications from www.brugadadrugs.org and advised strict avoidance of sodium channel blocking drugs. He was encouraged to have all siblings screened for Brugada syndrome if able. The patient was urged to seek medical attention for any further near or true syncope events.

## Discussion

Patients with BrS are sometimes unaware of their familial traits until they have symptoms that prompt immediate medical intervention when presenting with syncope or aborted cardiac arrest. Precipitating factors like fever, the use of certain sodium channel blocking drugs can trigger changes in the biophysiological properties of the cardiac sodium channels and cause shortening of the intra-epicardial dispersion of action potentials leading to life-threatening arrhythmias [1-4]. The symptoms occur more commonly in the early hours of the day due to increasing vagal tone.

There are three types of BrS based on the pattern of ST segments in the precordial leads V1-V3, corresponding T waves, and ST-segment terminal portion on ECG (Table 1).

**Table 1:** Brugada syndrome types

Types	
Type 1	ST segment elevations >2mm in V1-V3, with a coved pattern and inverted T waves, terminal ST portion gradually descending
Type 2	ST segment elevation >2mm in V1-V3, with a saddleback pattern, terminal ST portion >1mm and biphasic T waves
Type 3	ST segment elevation >2mm in V1-V3, with a saddleback pattern, terminal ST segment portion of <1mm

These patients have a positive provocative test with sodium channel blockers such as flecainide, ajmaline, or procainamide. About 20%-30% of the patients have positive genetic testing and a positive family history of sudden cardiac death in a first-degree relative less than age 45. 

BrP can present similarly with electrolyte imbalance such as hyperkalemia, hypercalcemia, hyponatremia, or from side effects of certain medications triggering asymptomatic Brugada type 1 or 2 ST elevations [5-9]. Treating the underlying cause can resolve ECG findings. Common underlying causes that can provoke BrP are included in Table 2.

**Table 2:** Underlying conditions that provoke Brugada phenocopy

Underlying conditions
Hyperkalemia
Hypercalcemia
Hyponatremia
Mechanical compression
Myocardial ischemia
Pulmonary embolism
Pericardial/myocardial diseases
Certain drugs: Class 1 anti-arrhythmic drugs, anesthetics (commonly propofol), tricyclic antidepressants, cocaine

In our patient, it was difficult to differentiate between BrS and BrP since he presented with a history of near syncope and sudden cardiac death of a first-degree relative and had ECG findings consistent with Brugada type 1 pattern on admission. Similarly in one case study from 2016, 10 international experts on BrS were given 6 ECGs each of clinically confirmed BrS and BrP [10]. Clinical history was not provided to the investigators. The observed diagnostic accuracy overall was 53±33%. This emphasizes the importance of accuracy in diagnosis as a misdiagnosis can cause significant mortality. Concise history taking to determine family history, medication history and identification of possible reversible causes are important. A diagnosis of BrP cannot be confirmed until all possible underlying causes have been eliminated and resolution of EKG patterns have been demonstrated [11-13].

We recommend that patients with BrS additionally be under continuous cardiac monitoring given the high risk for arrhythmia and sudden cardiac death. If arrhythmia inducing medications are identified they should be held immediately. Medications can be reviewed and compared to those that exist in online databases such as on www.brugadadrugs.org [14]. Electrolyte abnormalities should be identified and treated in a timely manner. Referral to an electrophysiology specialist should be done.

Our patient had coved ST elevations; Brugada type 1 pattern on arrival which converted to Brugada type 2 pattern on day 2 which normalized on day 3. Implantable cardioverter defibrillator (ICD) placement has not been shown to be necessary for those patients with BrP without sustained V-tach or other symptomatic arrhythmia. For those patients who experience life-threatening spontaneous sustained ventricular tachycardia, those who survived cardiac arrest or asymptomatic patients with inducible Brugada type 1 pattern ICD placement is necessary. 

## Conclusions

BrP and BrS are indistinguishable by ECG alone, further history taking and investigation are required to identify potential known underlying causes for BrP. BrP can only be diagnosed after these underlying causes have been eliminated and resolution of ECG findings are demonstrated. Patients with BrS do require close follow-up and counseling as with our patient, ICD placement is not needed in the absence of symptoms or life-threatening arrhythmia.
